# Pharmacodynamics of Finafloxacin, Ciprofloxacin, and Levofloxacin in Serum and Urine against TEM- and SHV-Type Extended-Spectrum-β-Lactamase-Producing Enterobacteriaceae Isolates from Patients with Urinary Tract Infections

**DOI:** 10.1128/AAC.02446-16

**Published:** 2017-04-24

**Authors:** A. Dalhoff, S. Schubert, A. Vente

**Affiliations:** a University Hospital Kiel, Institute for Infection Medicine, Kiel, Germany; b MerLion Pharmaceuticals, Berlin, Germany

**Keywords:** ESBL, finafloxacin, fluoroquinolones, pharmacodynamics, urine

## Abstract

The pharmacodynamics of finafloxacin, ciprofloxacin, and levofloxacin against extended-spectrum-β-lactamase (ESBL)-producing Enterobacteriaceae isolates were compared. Since quinolones lose activity in acidic media, and particularly in urine, their activities were tested in parallel under conventional conditions and in acidic artificial urine. For this purpose, TEM- and SHV-type ESBL-producing Escherichia coli and Klebsiella pneumoniae strains and their wild-type counterparts were exposed in a modified Grasso model to simulated concentrations of drugs in serum and urine following oral doses of either finafloxacin at 800 mg once a day (q.d.), immediate-release ciprofloxacin at 500 mg twice a day (b.i.d.), extended-release ciprofloxacin at 1,000 mg q.d., or levofloxacin at 500 or 750 mg q.d. The concentrations of the drugs in urine were fitted by compartmental modeling. Bacteria were cultivated in Mueller-Hinton broth (MHB) at pH 7.2 or 5.8 or in artificial urine at pH 5.8. Bacteria were counted every 2 h until 10 h and at 24 h; the areas under the bacterial-count–versus–time curves were calculated. It was found that finafloxacin eliminated all strains within 2 h under all the conditions studied. At all doses studied, ciprofloxacin and levofloxacin were highly active against wild-type strains in MHB at pH 7.2 but lost activity in MHB, and particularly in urine, at pH 5.8. Viable counts of ESBL producers were reduced for 6 to 8 h by 3 log_10_ titers, but the bacteria regrew thereafter. Ciprofloxacin and levofloxacin were almost inactive against the SHV producer grown in artificial urine. We conclude that pharmacodynamic models using artificial urine may mirror the physiology of urinary tract infections more closely than those using conventional media. In contrast to ciprofloxacin and levofloxacin, finafloxacin gained activity in this model at an acidic pH, maintained activity in artificial urine, and was active against TEM and SHV producers.

## INTRODUCTION

The bacterial etiology of urinary tract infections (UTIs) and acute pyelonephritis is dominated by Escherichia coli and Klebsiella spp. (1, 2). Most of the isolates from women with acute, uncomplicated UTIs (uUTIs) were fluoroquinolone susceptible, and extended-spectrum-β-lactamase (ESBL)-producing E. coli strains were almost absent (3–6). However, the prevalence of Enterobacteriaceae producing ESBLs of the TEM, SHV, and CTX-M types has increased significantly in patients with complicated UTIs (cUTIs) (7–13).

Although fluoroquinolone resistance was not correlated with ESBL production in general, it was associated with CTX-M production, whereas most of the TEM or SHV producers were fluoroquinolone susceptible (14, 15), probably because integrons have no major impact on the spread of ESBLs except for those of the CTX-M type (16–18). The coexistence of ESBL production and fluoroquinolone resistance may be due to an interplay between the prior use of β-lactams and fluoroquinolones (19). Chromosomal quinolone resistance mutations have been found in ESBL- or AmpC-producing Enterobacteriaceae isolated from humans, companion animals, and aquatic environments (20–25). Alternatively, plasmid-mediated quinolone resistance mechanisms, such as Qnr determinants, and ESBL production are coexpressed by the same plasmid (26–36). While some *qnr* genes are integrated into integrons, others are associated with transposons containing TEM-1-type β-lactamase genes (reviewed in reference 37).

Expanded-spectrum fluoroquinolones are recommended for the treatment of UTIs because of their pronounced activity against bacterial uropathogens and their high level of urinary excretion (1, 38, 39). However, levofloxacin and (particularly) ciprofloxacin have the potential for selecting methicillin-resistant Staphylococcus aureus (MRSA) (40, 41), and their use has been linked to MRSA infections (42). Therefore, expanded-spectrum fluoroquinolones should be “reserved for important uses other than acute cystitis” and are recommended as the first-line therapy for patients with uncomplicated pyelonephritis (43). This recommendation, however, is self-contradictory, since on the one hand, fluoroquinolones should not be used in uUTIs, which are caused by fully susceptible pathogens, but on the other hand, they should be used in cUTIs and acute pyelonephritis, which are frequently caused by ESBL producers that are multidrug resistant and/or resistant to expanded-spectrum fluoroquinolones.

The investigational fluoroquinolone finafloxacin is characterized by pronounced bactericidal activity against Gram-negative bacteria, including strains harboring plasmid-mediated quinolone resistance genes alone or in combination with chromosomal fluoroquinolone resistance mutations, as well as non-CTX-M-type ESBL-producing Enterobacteriaceae and Gram-positive bacteria, including MRSA and small-colony variants (44–46). The level of urinary excretion of finafloxacin is high (47), and its activity is impaired neither by an acidic pH nor by high concentrations of divalent cations, which prevail in UTIs (48–50). In contrast, the activities of expanded-spectrum fluoroquinolones are diminished under such growth conditions (48, 49). A phase II clinical study has demonstrated that pH activation of finafloxacin translated into the clinical arena. Patients treated with finafloxacin once a day (q.d.) for 5 days had higher, more-rapid, and more-sustained microbiological eradication and better clinical outcomes than those treated with ciprofloxacin administered twice daily (b.i.d.) for 10 days (50).

The aim of this study was to provide a rationale for the use of finafloxacin in the treatment of UTIs caused by TEM- and SHV-type ESBL-producing Enterobacteriaceae by comparison to two regimens each of ciprofloxacin and levofloxacin. Conventional or acidified Mueller-Hinton broth (MHB) and artificial urine were used as the media in order to simulate pathophysiological conditions as closely as possible.

## RESULTS

### Susceptibilities.

All strains tested grew well under all the experimental conditions studied. The activities of ciprofloxacin and levofloxacin for the ATCC reference strains grown in cation-adjusted MHB (CAMHB) at pH 7.2 were in agreement with the accepted ranges (Table 1). Finafloxacin gained activity in acidic CAMHB and maintained its activity in synthetic urine compared to that under standard test conditions in CAMHB, pH 7.2. In contrast, the activities of ciprofloxacin and levofloxacin were reduced in acidic CAMHB and were dramatically reduced in synthetic urine. The differences between MICs generated in slightly alkaline (pH 7.2) and acidic (pH 5.8) media were most marked for the test strain K. pneumoniae ATCC 700603, for which the MICs of ciprofloxacin and levofloxacin increased by 4 to 7 and 5 to ≥7 dilution steps, respectively.

**TABLE 1:** MICs of finafloxacin, ciprofloxacin, and levofloxacin against the indicator strains studied

Test strain^*a*^	MIC (mg/liter) of the following drug in the indicated medium^*b*^:								
	Finafloxacin			Ciprofloxacin			Levofloxacin		
	CAMHB		Syn. urine	CAMHB		Syn. urine	CAMHB		Syn. urine
	pH 7.2	pH 5.8		pH 7.2	pH 5.8		pH 7.2	pH 5.8	
Ec 1	0.06	0.03	0.06	0.03	0.06	0.5	0.06	0.25	1.00
Ec 2	0.06	0.03	0.06	0.03	0.12	0.5	0.06	0.25	2.00
Ec 3	2.00	0.50	1.00	2.00	8.00	16.0	1.00	8.00	32.0
Kp 1	0.125	0.03	0.125	0.03	1.00	2.0	0.06	0.25	8.00
Kp 2	2.00	0.50	2.0	0.25	4.00	32.0	0.50	16.0	≥64

a Ec 1, E. coli ATCC 25922 (wild type); Ec 2, E. coli ATCC 35218 (TEM-1); Ec 3, E. coli clinical isolate (TEM-3); Kp 1, K. pneumoniae ATCC 13883 (wild type); Kp 2, K. pneumoniae ATCC 700603 (SHV-18).

b CAMHB, cation-adjusted Mueller-Hinton broth; Syn., synthetic.

### Pharmacokinetic (PK) simulations.

The simulated drug concentrations deviated from the target concentration by 2.7% on average, which is within the accepted ranges of an equivalence interval (51). Growth controls in the absence of the study drugs grew almost equally well in the three media used (Table 2). Growth in CAMHB at pH 7.2 did not differ significantly from growth in CAMHB at pH 5.8, whereas the level of growth in synthetic urine was 2.4% lower, on average, than that in CAMHB, except for E. coli ATCC 25922 (mean, −9.2%).

**TABLE 2:** Comparative antibacterial activities of oral doses of finafloxacin, ciprofloxacin, and levofloxacin

Drug and strain^*a*^	AUBKC_0–24_^*b*^ (log_10_ CFU × h/ml) in the indicated medium^*c*^					
	Growth control		Pharmacokinetic simulation of growth in serum	Pharmacokinetic simulation of growth in urine		
				CAMHB		Syn. urine, pH 5.8
	CAMHB*, pH 7.2	Syn. urine, pH 5.8	CAMHB, pH 7.2	pH 7.2	pH 5.8	
FNX						
Ec 1	213.90	170.88	4.57	2.87**	2.87**	2.78**
Ec 2	190.23	180.18	4.63	2.76**	2.76**	2.70**
Ec 3	184.92	182.04	21.93	16.98	6.80	7.85
Kp 1	161.05	154.32	4.09	2.79**	2.79**	2.24**
Kp 2	179.36	158.15	19.99	8.24	2.77**	4.32
CIP-IR						
Ec 1	204.24	203.40	84.92	4.66	18.06	24.81
Ec 2	183.60	184.92	166.54	29.52	58.96	83.08
Ec 3	184.92	182.04	170.06	28.32	75.00	120.86
Kp 1	182.52	181.80	14.20	3.81	16.16	27.72
Kp 2	184.32	184.56	124.80	15.44	41.00	58.11
CIP-XR						
Ec 1	188.16	188.76	35.40	14.26	21.39	50.08
Ec 2	182.40	171.48	153.48	19.83	54.76	97.61
Ec 3	184.92	182.04	153.48	13.84	101.4	183.39
Kp 1	184.56	171.48	4.30	4.00	36.96	59.84
Kp 2	181.20	171.48	79.14	4.06	62.08	99.50
LVX 500						
Ec 1	211.60	184.32	3.75	3.07**	6.61	6.83
Ec 2	184.44	177.96	34.65	7.93	33.35	67.56
Ec 3	184.44	179.52	64.52	20.79	81.86	103.26
Kp 1	205.83	177.48	7.08	2.86**	2.98**	2.98**
Kp 2	184.08	181.20	90.54	8.20	17.16	83.43
LVX 750						
Ec 1	211.60	184.32	3.20**	3.03**	3.23	12.56
Ec 2	184.44	177.96	3.29	2.98**	2.99**	2.98**
Ec 3	184.44	179.52	50.54	13.86	62.13	82.83
Kp 1	205.83	177.48	3.29	2.98**	2.99**	2.98**
Kp 2	184.08	181.20	64.74	7.57	15.16	54.72

a Drugs: FNX, finafloxacin at 800 mg q.d.; CIP-IR, immediate-release ciprofloxacin at 500 mg b.i.d.; CIP-XR, extended-release ciprofloxacin at 1,000 mg q.d.; LVX 500, levofloxacin at 500 mg q.d.; LVX 750, levofloxacin at 750 mg q.d. Strains: Ec 1, E. coli ATCC 25922 (wild type); Ec 2, E. coli ATCC 35218 (TEM-1); Ec 3, E. coli clinical isolate (TEM-3); Kp 1, K. pneumoniae ATCC 13883 (wild type); Kp 2, K. pneumoniae ATCC 700603 (SHV-18).

b AUBKC_0–24_, area under the bacterial kill curve from 0 to 24 h.

c The test strains were incubated either in cation-adjusted Mueller-Hinton broth (CAMHB) adjusted to a pH value of 7.2 or 5.8 or in synthetic (syn.) urine (pH 5.8). *, the strains grew equally well in CAMHB at pH 7.2 and pH 5.8; **, differences in antibacterial activities under the different test conditions were not available, because viable counts of the test strains were reduced below the limit of detectability within the time between inoculation and the first sampling at 2 h.

**FIG 1:**
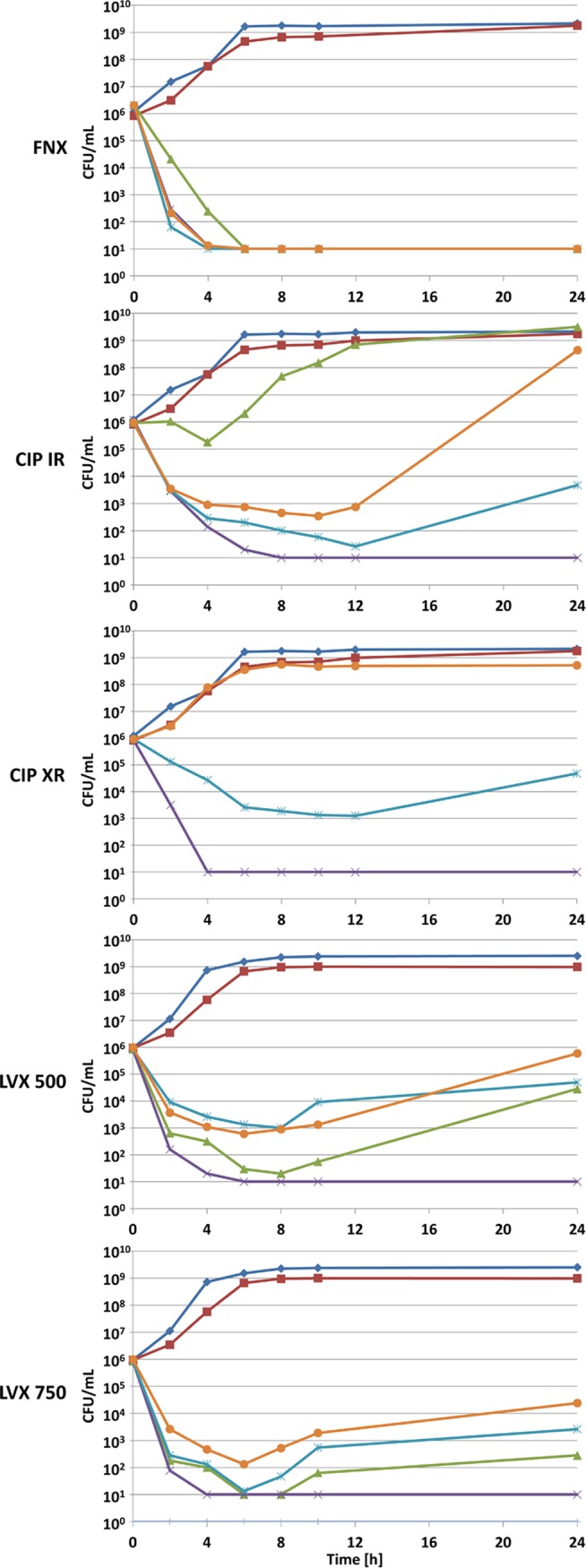
Pharmacokinetic/pharmacodynamic modeling of the bactericidal activities of finafloxacin, ciprofloxacin, and levofloxacin at various concentrations in serum or urine against a TEM-3-type ESBL-producing E. coli strain. FNX, finafloxacin; CIP-IR, immediate-release ciprofloxacin at 500 mg b.i.d.; CIP-XR, extended-release ciprofloxacin at 1,000 mg q.d.; LVX 500, levofloxacin at 500 mg q.d.; LVX 750, levofloxacin at 750 mg q.d. Symbols: dark blue diamonds, growth control in CAMHB; red squares, growth control in synthetic urine; orange circles, cyan starbursts, and purple multiplication signs, PK modeling of activities in urine by use of synthetic urine at pH 5.8, CAMHB at 5.8, and CAMHB at pH 7.2, respectively; green triangles, PK modeling of activities in serum by use of CAMHB at pH 7.2.

Time-kill curves with a TEM-3-type ESBL-producing E. coli strain exposed to the five regimens simulated under the four conditions studied are shown *pars pro toto* in Fig. 1. Simulated finafloxacin concentrations in urine and serum sustainably reduced viable counts of this strain below the limit of detectability within 4 h and 6 h, respectively, under all the conditions studied, irrespective of whether the pH was acidic or alkaline. For comparison, the two formulations each of ciprofloxacin and levofloxacin displayed comparable activity against this test strain only if the concentrations of the drugs in urine were simulated at pH 7.2 in CAMHB. Simulated concentrations of extended-release ciprofloxacin (ciprofloxacin XR) in serum were entirely ineffective, and those of immediate-release ciprofloxacin (ciprofloxacin IR) were almost ineffective. For both ciprofloxacin formulations, simulated concentrations in urine resulted in an initial reduction in viable counts for 12 h and in regrowth by 2 log_10_ titers thereafter in CAMHB at pH 5.8, whereas ciprofloxacin XR was ineffective in synthetic urine (pH 5.8) and ciprofloxacin IR resulted in a transient reduction in viable counts for the first 12 h and subsequent regrowth by 6 log_10_ titers. The simulated concentrations of both levofloxacin formulations tested in urine and serum were more effective than those of both ciprofloxacin formulations; however, they, too, were not as effective as finafloxacin. A sustained reduction in viable counts below the limit of detectability for levofloxacin was recorded only following exposure to simulated urine drug concentrations in CAMHB at pH 7.2. Under all other test conditions, an initial reduction of pathogens within 4 to 8 h was detected, but regrowth by 1 to 3 log_10_ titers occurred until 24 h.

Analogous data were obtained for the SHV-18-type ESBL-producing Klebsiella pneumoniae strain. Simulated concentrations of finafloxacin in urine reduced viable counts of this test strain most markedly in acidic media, as reflected by the low values for the area under the bacterial-kill–versus–time curve from 0 to 24 h (AUBKC_0–24_) (Table 2) compared to those for ciprofloxacin or levofloxacin (two regimens each). Likewise, the TEM-1-type ESBL-producing E. coli test strain was most markedly affected following exposure to simulated concentrations of finafloxacin in urine. High-dose levofloxacin was comparably active against this test strain, whereas the remaining regimens were moderately active.

## DISCUSSION

In agreement with previous studies, both discrete MIC endpoint determinations and PK simulations in an *in vitro* pharmacodynamic (PD) model have revealed that finafloxacin gains activity, but conventional fluoroquinolones, such as ciprofloxacin and levofloxacin, lose activity, in acidic media (44, 48–50). The dissociated activities of finafloxacin, on the one hand, and ciprofloxacin as well as levofloxacin, on the other hand, may be due in part to their zwitterionic structures resulting in different isoelectric points. The isoelectric points of ciprofloxacin, levofloxacin, and finafloxacin are 7.42, 6.8 (52), and 6.7 (53), respectively. These agents should be most active at the corresponding pH value, since the uncharged fraction only passes through prokaryotic and eukaryotic membranes, thus enhancing antibacterial activities against extracellular as well as intracellular bacteria. Dissociated *in vitro* activities (44, 48–50) and accumulation in eukaryotic cells (45) at acidic or alkaline pH values have been confirmed for finafloxacin and ciprofloxacin, respectively. However, the isoelectric points of finafloxacin and levofloxacin are almost identical, although the two agents are affected differently by pH changes. Therefore, differences in isoelectric points provide an adequate but not sufficient explanation for the dissociated activities of these agents. The nitrile substituent at C-8 of finafloxacin may increase its activity, too, since the nitrile group acts as a pseudohalogen. Another C-8 nitrile-substituted fluoroquinolone was considerably more active against Gram-negative and Gram-positive bacteria at an acidic pH than the hydrogen-substituted analogue and comparably active to halogen-substituted analogues (54, 55). In addition, finafloxacin is a poor substrate for efflux pumps in acidic as well as alkaline media (56, 57). Whether the high dipole moment of the nitrile group, the overall charge distribution, or both have an impact on the activity of finafloxacin should be investigated.

Apart from its activation in an acidic environment, finafloxacin differs from ciprofloxacin and levofloxacin by its higher activity against Gram-negative bacteria, including strains harboring plasmid-mediated quinolone resistance genes alone or in combination with chromosomal fluoroquinolone resistance mutations, as well as non-CTX-M-type ESBL-producing Enterobacteriaceae (44–46, 56). Therefore, the activity of finafloxacin, which is being studied clinically for the treatment of cUTIs or acute pyelonephritis (58), was assessed in an *in vitro* pharmacodynamic infection model against TEM- and SHV-type ESBL-producing strains in comparison to their susceptible counterparts. The tests were performed under experimental conditions that mimic the infectious focus most closely, i.e., cation-adjusted Mueller-Hinton broth adjusted to a pH of 7.2 or pH 5.8 and synthetic urine (pH 5.8) containing 11 solutes at concentrations found in a 24-h period in the urine of healthy men (59). Since urogenital infections comprise a broad spectrum of infectious entities, ranging from urosepsis or pyelonephritis to local infections, such as cystitis, prostatitis, or epididymo-orchitis, and exhibiting specific pathophysiological characteristics, and since planktonic bacteria are present in the urine in the course of any urogenital infection (60, 61), an antibacterial agent to be used for the treatment of UTIs should be active in an *in vitro* pharmacodynamic model simulating drug concentrations in serum and urine. Simulated serum PK profiles can be reasonably predictive of outcomes for infection sites where concentrations similar to those seen in plasma are achieved. This would probably also include cUTIs, where, in addition to achievable concentrations in urine, a tissue component is relevant to the infection (60, 61). If a uUTI is envisaged, then all the ciprofloxacin and levofloxacin regimens considered in this analysis would be predicted to provide maximum rates of kill and rapid eradication of susceptible wild-type bacteria in urine, while the TEM-1 β-lactamase producer and, in particular, the ESBL producers would be affected by ciprofloxacin or levofloxacin only marginally. The poor activities of ciprofloxacin and levofloxacin against the TEM-1 β-lactamase-producing E. coli strains studied may be due to the facts that transposon-associated, plasmid-mediated quinolone resistance genes of the *qnr* type—which reduce the activities of ciprofloxacin and levofloxacin, but not of finafloxacin–and TEM-1 β-lactamase genes may be coexpressed (37). These results are in good agreement with previously published data demonstrating that *in vitro*-simulated concentrations of ciprofloxacin IR and ciprofloxacin XR in serum were equally active against strains with MICs of ≤0.5 mg/liter only, whereas strains with higher MICs were not affected (62, 63). Likewise, neither the 500-mg nor the 750-mg levofloxacin regimen attained the pharmacodynamic targets against strains with elevated MICs of 0.25 to 0.5 mg/liter (64). The activity of finafloxacin in this *in vitro* pharmacodynamic model, however, was marked under all the experimental conditions studied. These data demonstrate that finafloxacin would be well suited for the treatment of uUTIs as well as cUTIs, irrespective of whether the uropathogens are highly susceptible or produce an ESBL of the TEM or SHV phenotype.

The impact of pH and divalent cations on the antibacterial activities of fluoroquinolones should also be considered for the interpretation of susceptibility testing. Based on EUCAST clinical MIC breakpoint definitions (susceptibility, ≤0.5 mg/liter; resistance, >1 mg/liter), even the two wild-type reference strains, which are susceptible to fluoroquinolones under routine test conditions at pH 7.2, are resistant in synthetic urine. Consequently, a large proportion of strains categorized as susceptible by standard susceptibility testing will shift to a borderline-susceptible or even a resistant category if tested under physiologically relevant conditions in synthetic urine. This hypothesis has been substantiated recently (65, 66). Even if ciprofloxacin and levofloxacin have favorable pharmacokinetic profiles, with relatively high concentrations in serum and very high concentrations in urine, thus compensating in part for the loss of activity in urine, pharmacodynamic surrogates such as the AUC (area under the concentration-time curve)/MIC and *C*_max_ (maximum concentration of the drug in serum)/MIC ratios will be much less favorable under physiologically relevant conditions. In contrast, the PK/PD surrogates for finafloxacin will remain almost unchanged.

Recent data from two phase II studies in patients with either uUTIs, cUTIs, or acute pyelonephritis indicate that the experimental data generated in this PK simulation study translate into the clinical arena. ESBL-producing pathogens isolated from patients suffering from complicated urinary tract infections or acute pyelonephritis were eradicated with finafloxacin in 91% of the cases and with ciprofloxacin in 0% of the cases within the first 3 days of treatment (50; A. Vente and M. Lückermann, unpublished data). Controlled clinical studies should address the hypothesis that finafloxacin is more active than ciprofloxacin or levofloxacin against TEM- or SHV-type ESBL-producing Enterobacteriaceae and may thus provide a therapeutic alternative to established fluoroquinolones.

## MATERIALS AND METHODS

### Bacterial strains and media.

Three β-lactam-resistant but fluoroquinolone-susceptible ESBL-producing strains and two susceptible wild-type strains were used: E. coli ATCC 25922 (wild type), E. coli ATCC 35218 (TEM-1 positive), an E. coli clinical isolate (TEM-3 positive), K. pneumoniae ATCC 13883 (wild type), and K. pneumoniae ATCC 700603 (SHV-18 positive).

Cation-adjusted Mueller-Hinton broth (CAMHB) at pH 7.2 or pH 5.8 (Oxoid GmbH, Wesel, Germany) was used as the standard medium. The synthetic urine, made up according to the method of Griffith et al. (59), contains 11 solutes, each at a concentration found in a 24-h period in the urine of healthy men; the final pH was adjusted to pH 5.8.

### *In vitro* model.

A one-compartment model according to the work of Grasso et al. (67) was used with slight variations. Briefly, this model consists of a central compartment into which the antibiotic-containing media were pumped via a programmable pump until the maximum concentrations of the drugs in serum or urine to be simulated were reached. Thereafter, antibiotic-free medium was pumped into the central compartment and was continuously eliminated in parallel to mimic half-life (*t*_1/2_) values. Control growth in the absence of antibiotics was monitored in parallel.

Mean concentration profiles in serum and urine following oral doses of 800 mg finafloxacin once a day (q.d.), 500 mg immediate-release ciprofloxacin twice a day (b.i.d.), 1,000 mg extended-release ciprofloxacin q.d., 500 mg levofloxacin q.d., or 750 mg levofloxacin q.d. were determined. Serum PK profiles were simulated in CAHMB at pH 7.2; urine PK profiles were simulated either in CAMHB at pH 7.2 or pH 5.8 or in synthetic urine at pH 5.8.

The concentrations of the three agents studied in serum and urine following oral administration were simulated based on published data (47–50, 59, 62, 67–72). Urine PK data were fitted from data on fractionated urinary recovery by compartmental modeling; this procedure allows the simulation of continuously fluctuating urine concentrations. The pharmacokinetic parameters simulated in the *in vitro* pharmacodynamic system are summarized in Table 3. Control growth in drug-free media was monitored in the same model under all the conditions studied. The actual drug concentrations were quantified in parallel to the determinations of viable counts by using a conventional cup plate agar diffusion test with a Bacillus subtilis spore suspension as the indicator organism.

**TABLE 3:** Pharmacokinetic parameters^*a*^ simulated in the *in vitro* pharmacodynamic system following oral administration of finafloxacin compared to other agents

Agent, dose^*b*^ (mg)	*C*_max_ (mg/liter)	*T*_max_ (h)	*t*_½_ (h)	Reference(s)
In serum				
FNX, 800 q.d.	11.0	1.0	10.0	37, 38
CIP-IR, 500 b.i.d	1.4	1.5, 13.5	5.5	43, 44
CIP-XR, 1,000 q.d.	2.3	3.0	6.5	45, 46
LVX, 500 q.d.	5.7	1.0	7.5	43, 46, 47
LVX, 750 q.d.	8.6	1.6	7.5	48
In urine				
FNX, 800 q.d.	180	2.0	7.0	17, 18
CIP-IR 500 b.i.d	450	1.5, 13.5	5.5	19, 20
CIP-XR, 1,000, q.d.	650	3.0	6.5	45, 46
LVX, 500 q.d.	580	1.5	9.0	43, 46, 47
LVX, 750 q.d.	870	2.0	9.0	48

a *C*_max_, maximum concentration of the drug in serum; *T*_max_, time to *C*_max_; *t*_½_, half-life.

b FNX, finafloxacin; CIP-IR, immediate-release ciprofloxacin; CIP-XR, extended-release ciprofloxacin; LVX, levofloxacin; q.d., once a day; b.i.d., twice daily.

Viable counts were determined immediately prior to the administration of the drug to the test system (0 h) and at 2, 4, 6, 8, 10, and 24 h; a 12-h sample was withdrawn immediately prior to the administration of the second 500-mg dose of ciprofloxacin IR.

The area under the bacterial-kill–versus–time curve from 0 to 24 h (AUBKC_0–24_), expressed as log_10_ CFU × h/ml, was calculated as described previously using the trapezoidal rule (73, 74). If discrete regrowth of a test strain was recorded at 24 h, but not between 10 and 24 h, the AUBKCs were calculated for the incubation period from 0 to 10 h, and the discrete data point at 24 h (calculated as log_10_ CFU per milliliter, divided by 2) was added to the AUBKC_0–10_ value.

In general, all the experiments were repeated once on a separate occasion. If the data differed, the higher values are reported.
